# Microstructure and mechanical properties of the Mg–Gd–Zn alloy prepared by sintering of rapidly-solidified ribbons

**DOI:** 10.1038/s41598-022-14753-2

**Published:** 2022-06-29

**Authors:** Wenbo Luo, Yanke Guo, Zhiyong Xue, Xiuzhu Han, Qinke Kong, Minghao Mu, Gaolong Zhang, Weimin Mao, Yu Ren

**Affiliations:** 1 Institute for Advanced Materials, North China Electric Power University, Beijing, 102206 China; 2 Beijing Institute of Spacecraft System Engineering, Beijing, 100094 China; 3 Tianjin Aerospace Electromechanical Equipment Research Institute, Tianjin, 300301 China; 4 School of Materials Science and Engineering, University of Science and Technology Beijing, Beijing, 100083 China

**Keywords:** Structural materials, Techniques and instrumentation, Engineering

## Abstract

Mg–15Gd–1Zn (wt.%) alloy was successfully prepared via the spark plasma sintering rapid solidification ribbons process. Microstructure investigation showed that the sintered alloys consisted of fine grains, the β_1_ phase, and long-perioded stacking ordered phase (LPSO). The sintering temperature and time have a significant effect on the microstructural evolution. A lower sintering temperature (430 °C ) was beneficial for obtaining finer grain sizes with less than 5 μm and a higher content of β_1_ phase with a content of 3–15 vol.% and a size-distribution of (10–600) nm. A higher temperature for a longer sintering time, 450–470 °C and 5–10 min, helpfully promoted precipitating the abundantly lamellar LPSO phase, and its content was 2–10 vol.% for LPSO phase with the width of (10–100) nm. The mechanical properties indicated that the fine grain size and supersaturated solid solution contributed at least 50% of the yield stress, and the residual contribution was related to the β_1_ phase and LPSO phase strengthening, which were based on their contents and the sizes.

## Introduction

Mg and its alloys have been paid more attention because of their high specific strength, good damping shock absorption, and easy machinability. They have been used in the fields of spacecraft, hydrogen tanks, wheel hubs, and other industrial products^1,2^. However, both the inferior strength and poor intrinsic plasticity still restrict the extensive application of alloys. The basic dilemma is the large anisotropy in dislocation activation-energy between the dominant slipping < *a* > dislocation at the basal plane and the secondary < c > dislocation (including < c > and < *c* + *a* >)^3,4^. The classic way to resolve the problem is regulating the microstructure of Mg alloys.

Several specific strategies were put forward to improve and optimize the microstructure of Mg alloys. Forming the appropriate lattice-defects in solid solution is the primary way to add alloying elements into the Mg matrix, such as Al, Zn, Gd, and other strengthening alloys^5,6^. Grain refinement is also a significant strengthening method for Mg alloys, because it obviously impedes the movement of dislocations around the grain boundaries (GBs)^7,8^, the smaller grains, the more strengthening of Mg matrix^9^. In addition, the appropriate phase boundaries (PBs) coordinate the movement of various kinds of dislocations, and therefore, it is always an unremitting pursuit with introducing single or multiple strengthening phases with fine sizes in Mg matrix. When the strengthening second-phases were precipitated from the Mg matrix (solid solution), the different kinds of dislocation energy gaps could be significantly decreased along with the propagation of dislocations; thus, both strength and plasticity would be improved^10,11^. However, each of the above methods has a limited strengthening effect, and the three methods must be combined to achieve the superb mechanical properties of Mg alloys.

Both GBs and PBs could simultaneously increase when adopting the conventional thermal–mechanical plastic forming process and heat treatment process in Mg–Gd-Y–Zn–Zr series alloys^12–14^, which is a kind of vital high-strength alloy^12,13,15^. The multiscale hierarchical crystal structure was then achieved, which has been studied in face center cubic (*fcc*) and body-centered cubic (*bcc*) lattices^16^. The rolled Mg–8.2Gd–3.8Y–1.0Zn–0.4Zr (wt.%) alloy, its strengths increased by about 200%, and the true strain to failure also increased by 110%, when contains both submicron grains and nanosized precipitate phase^17,18^. Recently, two second-phases or multiple strengthening second-phases were also developed for high properties Mg alloys. For some Mg–RE–Zn alloys (RE, rare earth elements), the microstructure composed of α-Mg + lamellar LPSO phase + β′ phase. Similar the rolled and aged Mg–8.2Gd–3.8Y–1.0Zn–0.4Zr (wt.%) alloy, the ultimate strength is approximately 450 MPa with an moderate elongation to fracture of 10%^17^. And the extruded Mg–10.3Zn–6.4Y–0.4Zr–0.5Ca (wt.%) alloy, shows the strengths larger than 400 MPa and an elongation of 4%, in the condition of containing nanosized W phase and β_2_ phase particles^18,19^. However, due to easy segregation of alloying elements during solidification, the kind of second-phase was still hard to control in the conventional Mg alloy preparation process. For these Mg-RE intermetallic strengthening phases, controlling its structure and distribution was a relative long process (solid-solution heat-treatment, and then aging treatment). Recently, the optimizing microstructure were extensively studied basing rapid solidification (RS) method, especially for the low temperature sintering and forming with a severe plastic deformation (SPD). Gerardo Garces et al. adopted equal channel angular pressing (ECAP) method to prepare high strength Mg_98.5_Y_1_Zn_0.5_ alloy containing LPSO phase^20^, showing significant strengthening effect, and its yield stress was 300–364 MPa, and the elongation of 3–16%. In addition, Daria Drozdenko et al.consolidated the RS Mg-Y-Zn ribbon using hot-extrusion method^21^, and its yield stress was 362 and a elongation of 18.2%, the high mechanical property was mainly because fine grains with bimodal microstructure and LPSO phase.

It is well-known that, the β′ phase, β_2_ phase, β phase, and β′′phase precipitates are important strengthening phases in Mg-RE-Zn alloys. The β′ phase with an orthorhombic structure (Mg_7_RE) is metastable. The β_2_ phase (MgZn_2_) had a hexagonal structure, mainly formed in the former treatment process and existed in Mg-RE or Mg-RE-Zn series alloys^22^. Moreover, the β phase with a cubic structure (Mg_5_RE, a = 2.23 nm) was a stable equilibrium phase, the metastable β′′phase has a *D0*_*19*_ structure (Mg_3_RE, a = 0.64 nm). Among these phases, the β′ was the most expected strengthening precipitated phase. Another kind of β series phase, the metastable β_1_ phase with an *fcc* structure (Mg_3_RE, a = 0.73 nm), has rarely been studied in strengthening Mg alloys. The β_1_ phase was regarded as a harmful second phase in Mg-RE-Zn alloys, this is because RE atoms (like Gd) easily segregate on the grain boundaries and then grow up to a bulk or netlike shape phase, which is barely eliminated in the latter plastic deformational and heat-treatment process, and therefore deteriorates the mechanical properties of Mg alloys^22,23^.

To take advantage of the strengthening effect of the β_1_ phase, we developed a method based on the low temperature sintering (spark plasma sintering, SPS) rapid solidification (RS) strip process^24^, to achieve regulating the precipitation behavior of supersaturated solid solution (SSSS) and controlling the β_1_ phase size. Using this method, a multiple microstructure with fine Mg grains, LPSO phase, β_1_ phase, and β′ phase was successfully prepared^25^. SPS conducts a pressure and pulsed current assisted sintering process and belongs to a more general class of electric current activated sintering (ECAS) techniques^26–28^, the fine grains and appropriate second-phases’ sizes and contents could be controlled with different sintering parameters.

In this work, the microstructure evolution of sintered Mg-Gd-Zn alloy was studied, and the microstructure evolution containing LPSO phase and the β_1_ phase was investigated in detail. The relationship of mechanical properties and multiple precipitated second-phases was in-depth discussed in this study. This would provide a feasible approach for achieving high property Mg alloy with high strength and good toughness.

## Experimental

**Figure 1:**
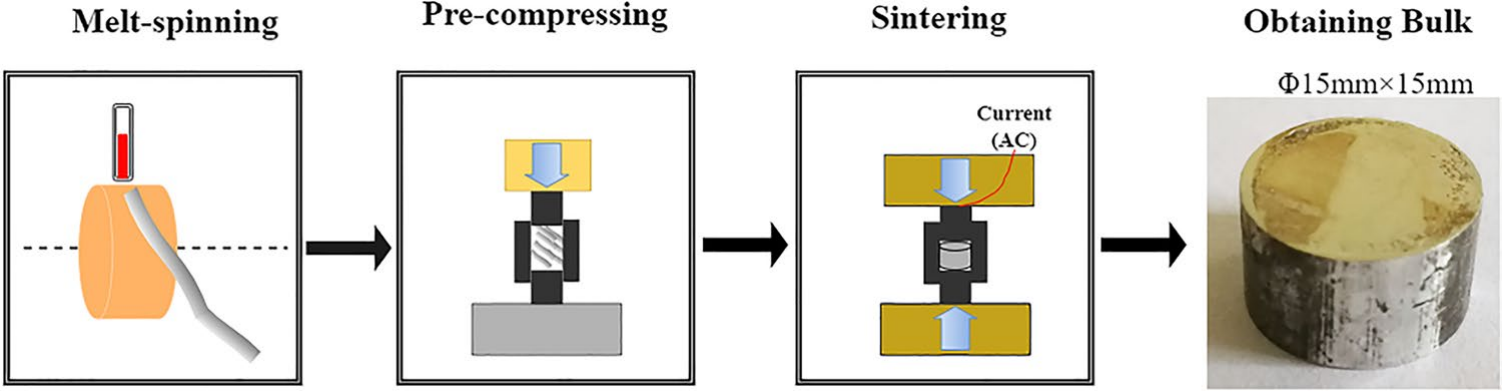
Schematic diagram of preparing the sintering RS Mg–Gd–Zn bulk.

The nominal composition of studied alloy was Mg–15Gd–1Zn (wt.%). Figure 1 shows the schematic diagram of sample preparation. First, the alloy was prepared with the raw materials: Mg (99.95 wt%), Zn (99.95 wt%), and Mg-Gd alloy (30 wt%), were melted at 730 °C and then poured into an ingot with the air protection of CO_2_ and SF_6_ (the volume ratio was approximately 99:1). Subsequently, the ingot underwent a heat-treatment at 500 °C for 12 h to reduce element segregation. Them, the ingot was remelted via induction heating to prepare a rapid solidification (RS) ribbon of continuous length (not shorter than 1000 mm) with a plane flow casting after removing the outer layer of the ingot. The heating temperature was kept at 710 °C , the copper-roller speed was about 80–90 r·s^−1^, in a N_2_ atmosphere (∼5 kPa). The related ribbon-preparation parameters were described in our previous study^25^. The obtained ribbons were conserved in liquid nitrogen. The RS ribbons were subsequently cut and filled into a steel-mould, and pre-compressed with a hydropress at a 20 MPa pressure for 3 min for each sample. The consolidation was conducted by a spark plasma sintering (SPS) system (Fuji DR. series). The sintering temperature was 430–470 °C, the holding time was 3–10 min, and the sintering pressure was 40–50 MPa. The detailed sintering parameters shown in Table 1. The original size of each sintering bulk sample is about Φ 15 mm × 15 mm.

**Table 1:** Relative density of the various sintering Mg alloy bulks.

Sintering NO	Temperature (^o^C)	Pressure (MPa)	Time (min)	Relative density
1	430	40	3	0.927 (< 0.98)
2			5	0.997
3			10	0.997
4		45	3	0.988
5			5	0.994
6			10	0.999
7		50	3	–
8			5	0.997
9			10	1.004 (melted)
10	450	40	3	0.945(< 0.98)
11			5	0.999
12			10	1.008 (melted)
13		45	3	0.994
14			5	0.995
15			10	0.999
16		50	3	–
17			5	0.998
18			10	1.010 (melted)
19	470	40	3	0.955(< 0.98)
20			5	–
21			10	1.009 (melted)
22		45	3	0.993
23			5	0.995
24			10	1.009 (melted)
25		50	3	–
26			5	0.998
27			10	1.010 (melted)

The density of the sintering alloy was based on the Archimedes p rinciple, and the relative densities are shown in Table 1. The origin density data was shown in Supplementary Table S1. There were 27 possible kinds of sintering conditions, and 23 samples were sintered. Among the sintered samples, there were 8 samples that showed the inapposite densities due to the low relative densities, only about 0.93–0.95, at the sintering process of 430 °C −40 MPa-3 min, 450 °C −40 MPa-3 min, and 470 °C −40 MPa-3 min. Similarly, four samples had actually remelted during the sintering process, at the sintering processes of 430 °C −50 MPa-10 min, 450 °C −40 MPa-10 min, 450 °C −50 MPa-10 min, 470 °C −40 MPa-10 min, and 470 °C −50 MPa-10 min, and therefore, the latter investigation mainly focused on those successful sintering bulks, for their relative densities located in the range of 0.980–0.999.

The compressive samples with dimensions of size of Φ 4 mm × 8 mm, were cut from the center of sintering bulks using an Electron Discharge Machining (EDM) and the sample surfaces were polished. The compressive tests were conducted using an Instron 3384 universal testing machine, at room temperature with a strain rate of 10^–3^ s^−1^. Testes for each sample were repeated for three times.

X-ray diffraction (XRD, Rigaku Ultima IV 3 KW, Cu-Kα radiation), and scanning electron microscopy (FE-SEM, Zeiss Auriga-EVO 18 field-emission, 0–20 kV), which was equipped with an energy dispersive X-ray spectrometer (EDS) system, are used for the microstructure characterization and the phase identification. The samples for SEM observation were etched in a solution of 4 vol% nitrate alcohol after polishing the surfaces. In addition, a transmission electron microscope (TEM, Tecnai G2 F30, 300 kV) was also used to investigate the second-phase characterization in detail. The TEM foils were prepared first mechanically polished to ∼50 μm, punched into discs 3 mm in diameter, and then further reduced using a Gatan plasma ion polisher. The statistic sizes of grains and particles are analyzed within the SEM and TEM images. The grains size was measured with intercept counting method (ASTM E112-96), the grain size NO. (G) was 12 and 12.5 (the corresponding average grain sizes were about 5.6 and 4.7 μm). For further determining the grains evolution at different sintering temperature, ~ 300 grains were random taken and the then average-size were counted in each experiment. For the sizes of β_1_, the lengths of 300 random particles were counted, and then the average size was calculated, the detailed data was shown in Supplementary Table S2. The mean width of LPSO phase was obtained when counting about 200 slices taken from TEM images. 

## Results and discussion

### The microstructure evolution during sintering processes.

**Figure 2:**
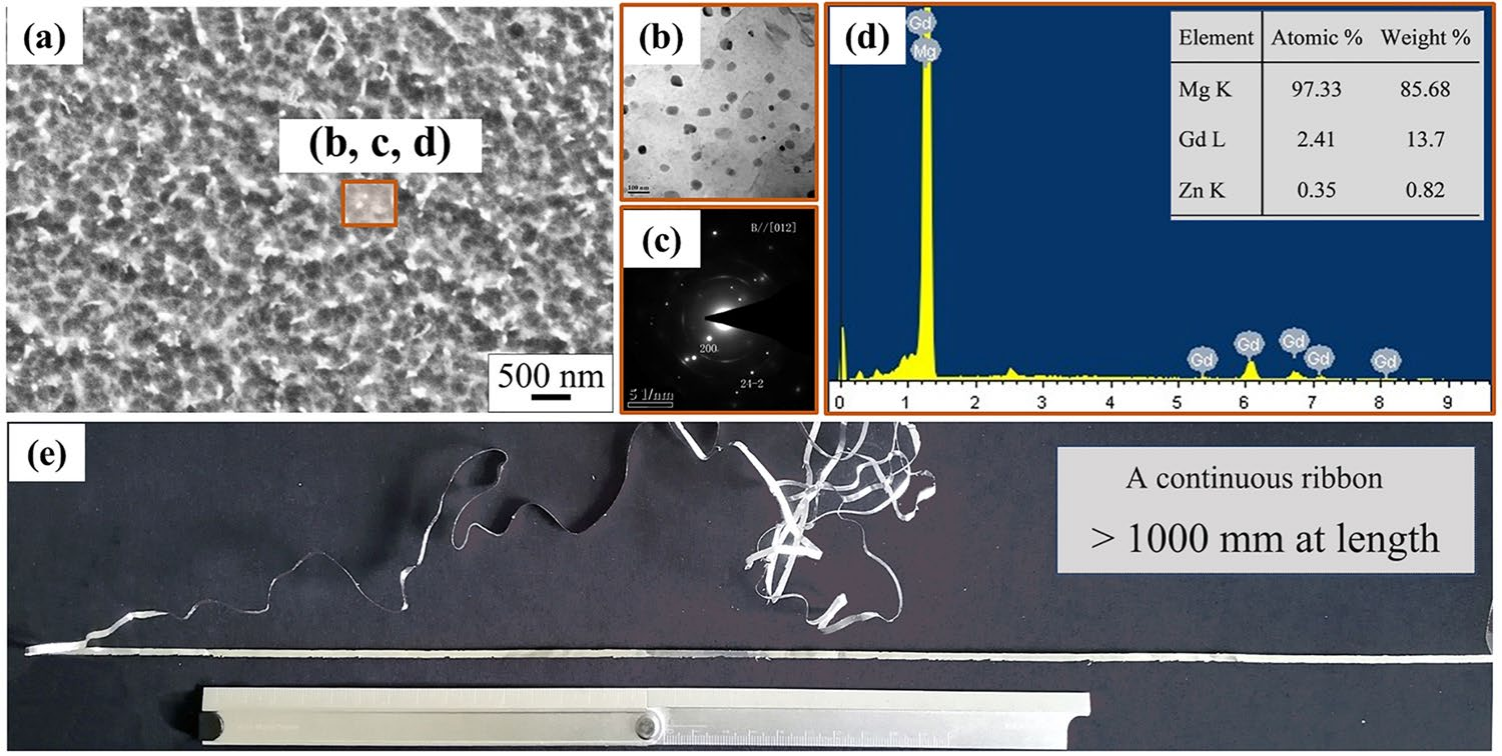
The RS Mg–15Gd–1Zn ribbon: (**a)** the SEM image showing very fine grains; (**b**,**c)** the bright-field TEM image and the corresponding selected area electron diffraction (SAED) pattern; (**d)** the EDS analysis of SSSS Mg matrix; (**e)** the microstructure of the RS ribbon.

Figure 2 shows the macrostructure and microstructure of the Mg-15Gd-1Zn RS ribbon. The length of the ribbons was longer than 1000 mm, with a width of 5 mm and a thickness of 56 ± 5 μm. The microstructure of the RS ribbons consists of fine grains and small second phase particles. The grain-sizes were mostly less than 300 nm, most second phase particles were distributed on the grain-boundaries, and their sizes were less than 100 nm. TEM analysis shows that the second-phase particles were (Mg, Zn)_3_Gd (β_1_) phase with a face-centered cubic (*fcc*) structure.

In addition, the EDS results indicate that most Gd and Zn atoms were fixed in the α-Mg matrix solid solution. Their contents consisted of (2.41 ± 0.3) at.% Gd (~ 14 wt.%), (0.35 ± 0.1) at.% Zn (~ 0.62 wt.%) and balanced Mg, and the values were much higher than those of the equilibrium densities of Gd and Zn in Mg alloy at room temperature (both the values were approximately 0.01%). The high densities of alloying atoms in Mg-based solid solution are crucial to controlling the precipitation of multiple second-phases.

**Figure 3:**
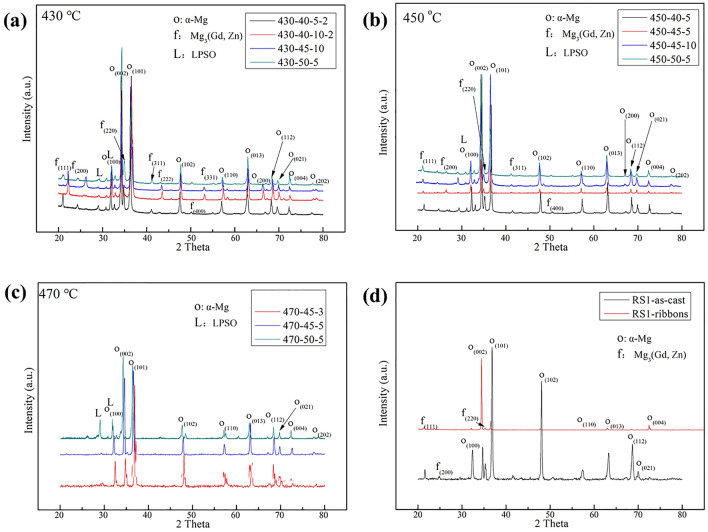
XRD patterns of the Mg–15Gd–1Zn alloy under different sintering conditions: (**a)** at 430 °C; (**b)** at 450 °C; (**c)** at 470 °C; (**d)** the RS ribbon and its as-cast ingot.

Figure 3a–c shows the XRD patterns of various phases in the samples sintering at 430–470 °C. The sintered bulks have been determined to be consisted of α-Mg, LPSO, and β_1_ phases, comparison with that of its as-cast ingot and RS ribbon, as shown in Fig. 3d. The phases of the as-cast alloy and RS Mg–15Gd–1Zn ribbon consist of α-Mg and β_1_ phase (Mg_3_(Gd, Zn)). The orientation-relationship of phases has a significant change with sintering at various temperatures, sintering time, and pressures.

For the α-Mg phase, the two main peaks (lattice-plane) are (002)_α_ and (101)_α_. (101)_α_ was the fastest-growing peak during the following sintering processes: 430 °C sintering for 10 min, and 450–470 °C with a pressure of 40–45 MPa. It is well-known that the (101)_α_ plane is the close-packed basal plane, and it is also the main peak in most Mg alloys^29–31^. This result indicated that the long sintering time and lowr sintering pressure were more beneficial to the α-Mg phase equilibrium transformation. On the other hand, at higher sintering pressures, such as 50 MPa, (002)_α_ was still the main orientation. This indicates that high pressure was convenient to maintain the metastable Mg crystal structure. In addition, their intensities of (102)α and (110)α decreased with increasing sintering pressure. In contrast, the other peaks had little effect with the change in sintering time and sintering pressure, such as (013)α, (112)α, and (021)α. The phase-evolution of β_1_ phase was also investigated, the primary main-peak was (111)_β1_ in the RS ribbon. While the main-peak changed to (220)_β1_ and its intensity also increased slightly sintering at 430–450 °C. Additionally, the growth of the (222)_β1_ plane was obvious with prolonging the sintering time, as shown in Fig. 3a. The other crystal planes grew slightly, such as the (200)_β1_, (311)_β1_, and (222)_β1_. When it sintered at higher temperature, 470 °C, however, the β_1_ phase was not detected. This was probably because the lamellar LPSO phase obviously grew up, and therefore impeded the growing-up of the β_1_ phase.

The formation of the LPSO phase was mainly related to the changing of crystal planes of (100)_α_ and (002)_α_, due to the close orientation relationship between the LPSO and α-Mg phases. The intensities of the LPSO phase gradually increased with the prolonging of sintering time and rising of sintering temperature. At a temperature of 470 °C, the intensity of the LPSO phase significantly increased.

During the sintering process, the peaks change of β_1_ phase is coincidence with the orientation relationship between β_1_ phase and Mg matrix, (-112)_β1_//(210)_α_ (the prismatic plane), [110]_β1_//[001]_α_^22,24^. The rearrangement of atoms from SSSS leads to forming the close-packed planes of Mg and β_1_ phase during the sintering process, as is LPSO phase. TheLPSO phase was a special hexagonal crystal, and the orientation relationship between 14H and a-Mg is that (0001)_LPSO_//(0001)_α_ and[0–110]_LPSO_ //[1–210]_α_^31^. Its formation is closely related to the Zn-Gd clusters and atom arrangement over a long period. It has been proved that the LPSO phase is easily formed when the temperature is higher than 400 °C with a long heat-treatment time in the Mg–Gd/Y–Zn(Cu) alloys^31–34^. The content of Gd reduced from 2.4 at.% (about 14 wt.%) to 2.0 at.% (12 wt.%) in the sintering Mg-15Gd-1Zn bulks. The substitutable Gd atoms at (0001)_α_ plane and nearby Zn atoms in SSSS provide favorable conditions for the formation and growth of the LPSO phase. Both high sintering pressure and long-time are beneficial to the growing-up of the lamellar LPSO phase. More lamellar LPSO phases are found under sintering for 10 min at 430 and 450 °C, as shown in Fig. 3a,b.

**Figure 4:**
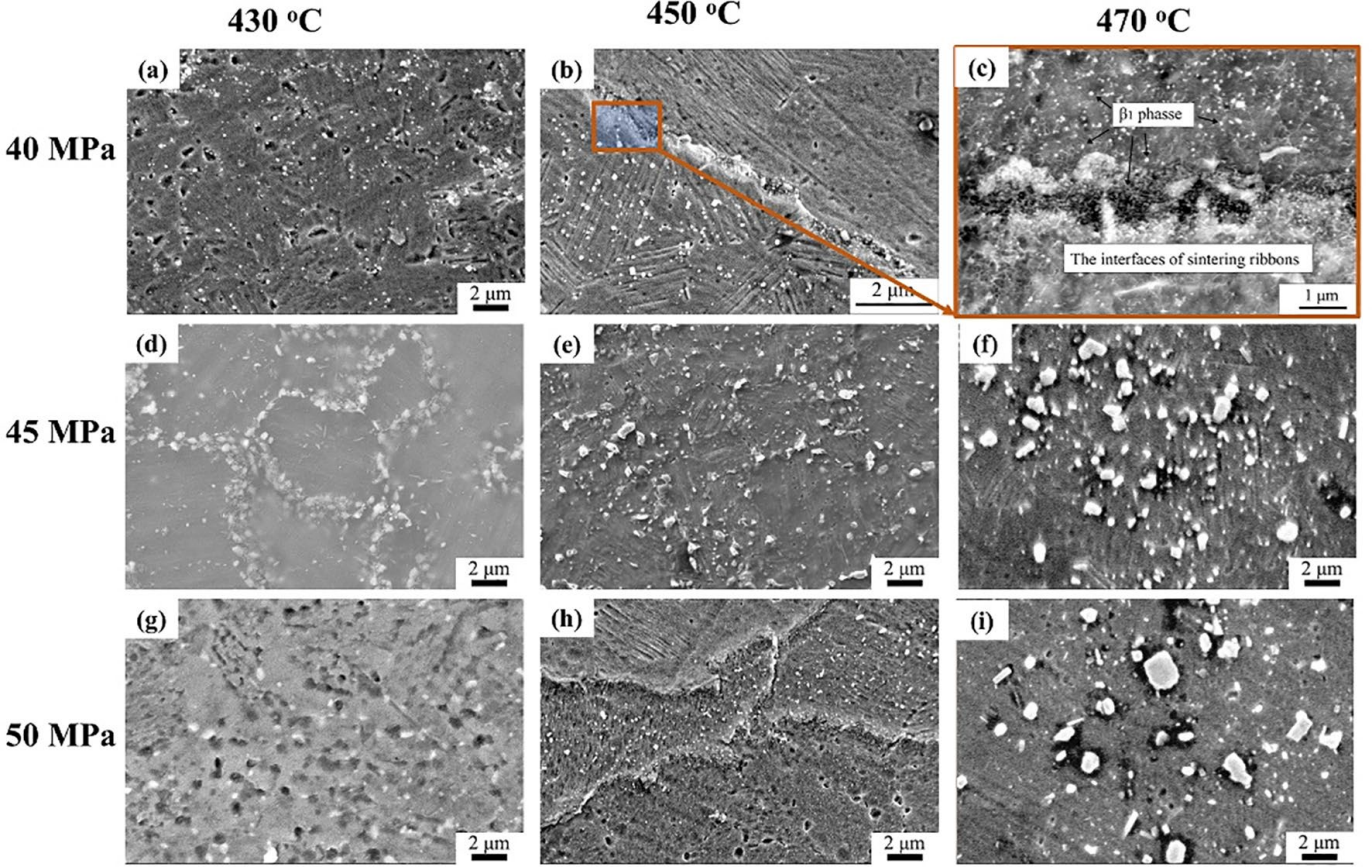
SEM images of sintered Mg-15Gd-1Zn alloy at various sintering pressures (40–50 MPa) and temperatures (430–470 °C): (**a**,**b)**, and (**c)** for 40 MPa; (**d–f)** for 45 MPa; (**g–i)** for 50 MPa, each time of sintered bulk was 5 min.

Figure 4 shows the SEM morphology of sintered Mg-15Gd-1Zn bulks at various temperatures (430–470 °C) and different pressures (40–50 MPa). Both the grains and second-phases grew up slightly during the sintering process. The second phases, including β_1_ phase particles and LPSO phases, significantly changed in size and distribution. Most of the β_1_ phase particles are located on grain boundaries and the Mg matrix. High pressure has a few effects on the size of β_1_ phase particles, which are the smallest at a pressure of 40 MPa. They increase by approximately 14% and 57% with an increase in sintering pressure (at each sintering temperature), as shown in Fig. 4a,d,g, and Fig. 4b,d,h.

At each sintering pressure, the temperature significantly affected the grains and β_1_ phase particles. With the sintering temperature rising from 430 to 470 °C, the mean grain sizes increased slightly: (4.6 ± 1.1) μm, (5.1 ± 1.5) μm, and (5.8 ± 1.5) μm. In addition, the number of β_1_ phase particles increased slightly. Their sizes are approximately (88–139) nm at 430 °C, (104–171) nm at 450 °C, and (221–293) nm at 470 °C.

The lamellar LPSO phases formed in the sintering process and were distributed in the matrix, as shown in Fig. 4e,f. The effect of higher sintering temperature on the formation of the LPSO phase is greater than the sintering pressure. The plentiful LPSO phase is precipitating with a lamellar shape, and the phase grew slightly with sintering time increased to 5 min.

**Figure 5:**
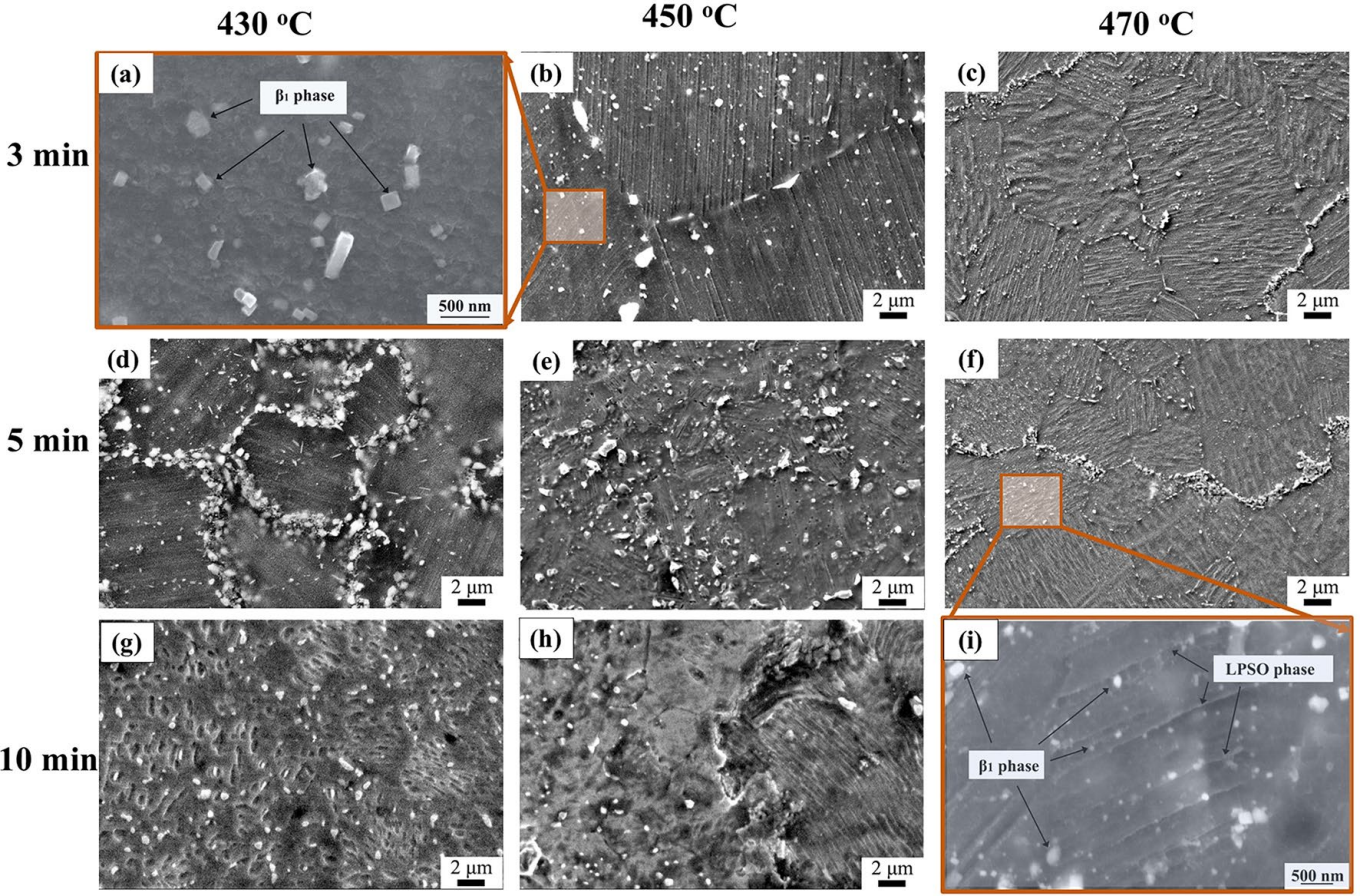
SEM images of sintered Mg–15Gd–1Zn alloy at various sintering times (3–10 min) and temperatures (430–470 °C): (**a–c)** for 3 min; (**d–f)** for 5 min; (**g–i)** for 10 min, each pressure of sintering bulk was 45 MPa.

**Figure 6:**
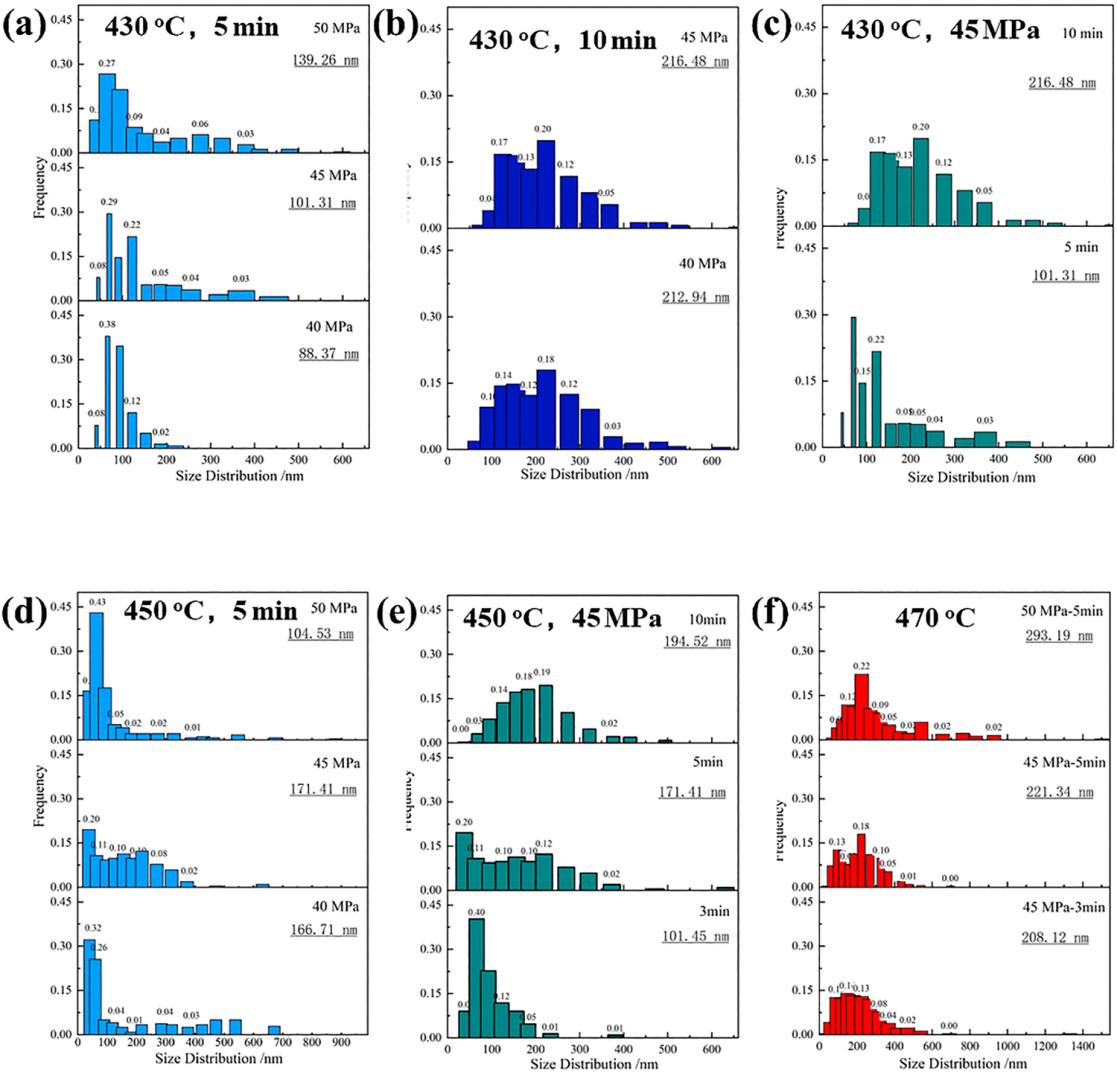
Size-distribution of β_1_ phase particle at various sintering conditions: (**a–c)** at 430 °C; (**d**,**e)** at 450 °C; (**f)** at 470 °C, the mean size was inserted in the corresponding figure.

Figure 5 shows the morphology of those bulks at various sintering times (3–10 min) and different temperatures (430–470 °C). Especially for those β_1_ phase particles, the results indicated that a short sintering time, 3–5 min, is beneficial to maintain the small sizes of both grains and second-phases. The sizes of most of the β_1_ particles were less than 200 nm. With the sintering time prolonged from 5 to 10 min at 430–450 °C, the number of β_1_ phase particles and LPSO phase increased. The detailed size distribution of the β_1_ phase is summarized and shown in Fig. 6, and the original statistical data are displayed in Supplementary Table S2.

At 430 °C, the size of most of these particles was less than 200 nm at 430–450 °C, as shown in Fig. 5a–e. The smaller particle sizes were less than 100 nm. Its ratio was approximately 78% under sintering time of 5 min. With an increase in sintering pressure from 40 to 50 MPa, the smaller particles slightly decreased to about 57% at 45 MPa, and 61% at 50 MPa. Meanwhile, those large β_1_ phase particles, which were larger than 200 nm, also increased by approximately 20%, too, showing a bimodule size distributing. When the sintering time was prolonged to 10 min, the size of the particles increased, and the content of the smaller particles was only about 20%, while the larger particles were approximately 45%, meanwhile, approximately 45–40% of particles were located in the range of 100–200 nm.

At 450 °C, the size distribution had similar behaviors at a temperature of 430 °C. However, it was noted that the particles were finer when kept at 50 MPa pressure, more nucleation occurred at the high sintering temperature, and more particles were formed during the dissolving of unstable SSSS. With the sintering temperature rising to 470 °C, β_1_ phase particles grow up obviously, and the size of most of them is in the range of 100–600 nm (approximately 90%). While sintering for 3 min at a higher temperature, 470 °C, 90% of the particles are located in the range of 100–400 nm, and with time prolonged to 5 min, the particles significantly increase and distribute with bimodule sizes of 150–300 nm and 450–650 nm.

The above investigation showed that the sintering temperature is the foremost factor controlling the size of the phase, and high pressure at higher temperatures could improve the formation of the β_1_ phase nucleus, which was convenient for forming smaller β_1_ phase particles.

**Figure 7:**
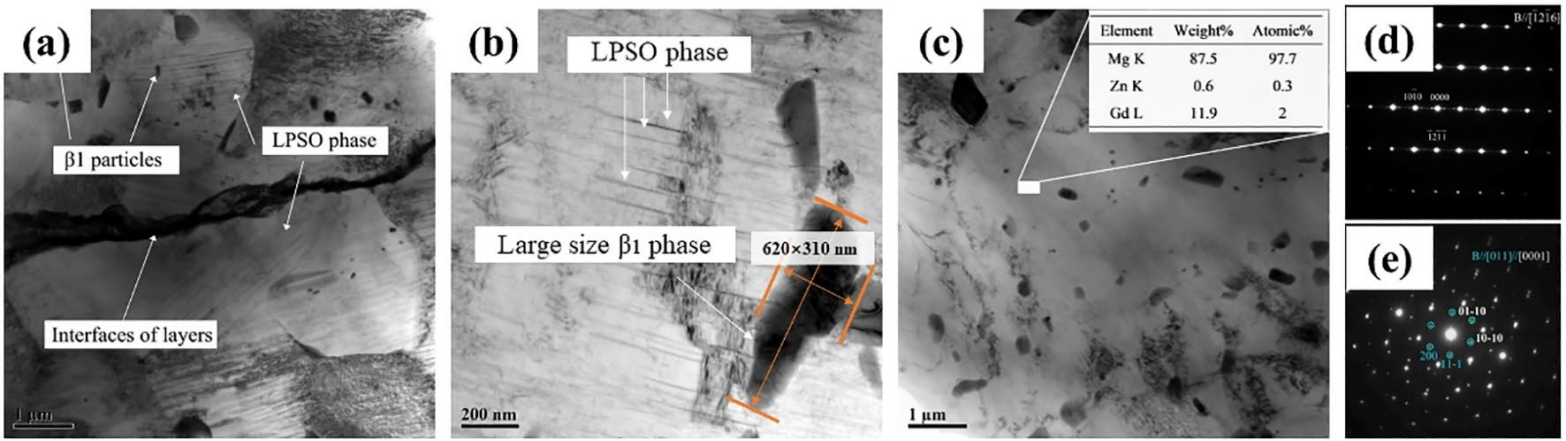
TEM images of SPSed Mg-15Gd-1Zn bulk under the sintering condition of 450 °C −40 MPa-5 min: (**a**,**b)** displaying the lamellar LPSO phase formed during the sintering process; (**c)** the β_1_ phase particles, (**d**,**e)** showing the selected area electron diffraction (SAED) patterns of LPSO phase and β_1_ phase, respectively, the beam was parller to^12–16^ and [0001] (011), the white indices were for the Mg matrix with a *hcp* structure, and blue indices were for β_1_ phase with a *fcc* structure.

Figure 7 shows the BF TEM images of the second-phases. The results that the LPSO phase directly forms from the Mg matrix and the interfaces of ribbon layers. The width of the phase was very fine, and the orientation was invariably the same in each grain, as shown in Fig. 7a,b. The larger β_1_ phase particles with 620 nm length and 310 nm width was found to be located around the LPSO phase. Figure 7c shows the fine β_1_ phase particles and Mg matrix containing approximately 2.0 at.% (12 wt.%) Gd and 0.3 at.% (0.6 wt.%) Zn. Furthermore, the contents of both second-phases were estimated to be about 2–10 vol.% for the LPSO phase and 3–15% for the β_1_ phase particles in the sintered bulk.

**Figure 8:**
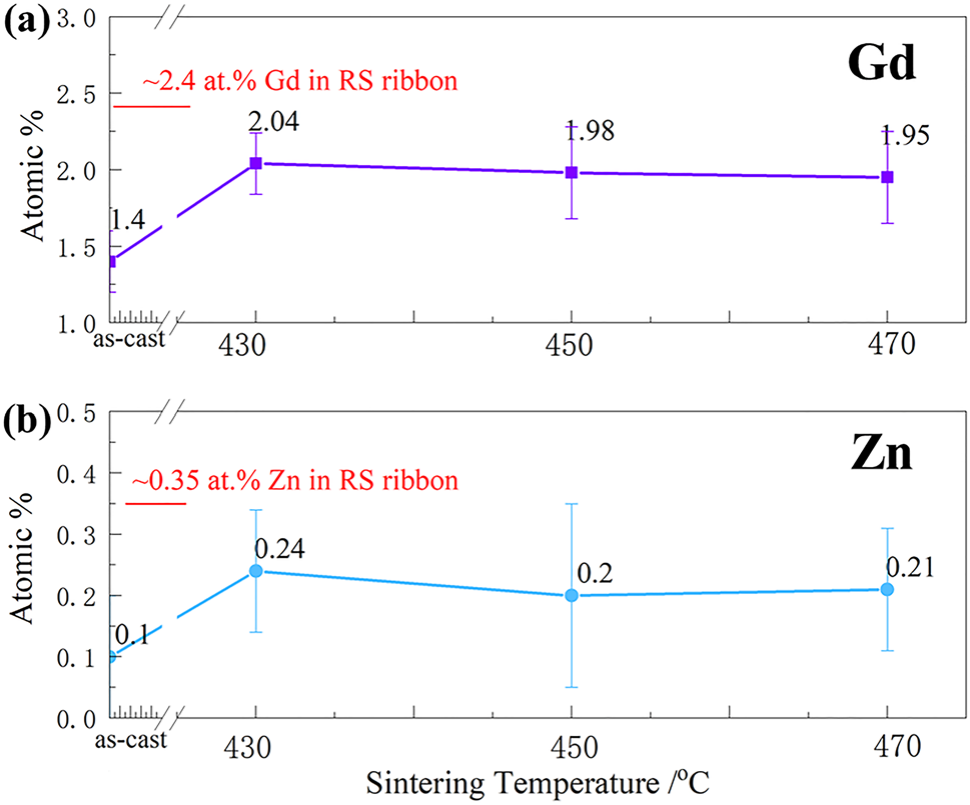
Gd and Zn contents of the solid solution in sintered bulks at different temperatures: (**a)** Gd; (**b)** Zn.

Figure 8 shows the content of Gd and Zn atoms in Mg-15Gd-1Zn alloys under different states. During its as-cast ingot, the contents of Gd and Zn were 1.4 at.% (8.5 wt.%) and 0.1 at.% (0.3 wt.%). With rapid solidification and sintering of different temperatures, the contents of solute atoms are still kept to high levels, about 2 at.% for Gd (12 wt.%) and 0.2 at.% (0.5 wt.%) for Zn in all the sintered bulk.

### The mechanical behaviors of sintered bulks

**Figure 9:**
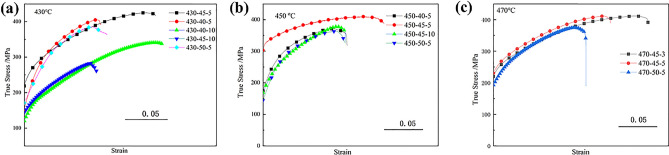
True stress–strain curves of sintered Mg–15Gd–1Zn alloys: (**a)** at 430 °C; (**b)** at 450 °C; (**c)** at 470 °C.

Figure 9 shows the true stress–strain curves of these bulks obtained from various sintering conditions. These yield strength and true strains were affected by both the sintering temperature and sintering time. The yield strength values of the samples sintered at 430 °C showed more significant fluctuations than those of 450–470 °C. At lower temperatures, a longer sintering time is beneficial for the true strain of the sintered alloy. The true strain increased from 10% at 5 min to 16% at 10 min under the sintering temperature of 430 °C. With the rising temperature, the yield strengths of the sintered alloy had increased by about 25% for those of the lower temperature and long sintering time samples. The ultimate strength also increased with rising sintering temperature (at 450, 470 °C). The short sintering time was better at a higher temperature for the plasticity (true strain). The true strain reached 16% and 22% when the sintering time was 3 min at 450 °C and 470 °C, respectively. Table 2 shows the values of stresses and true strain to failure for the sintered bulks. Their yield strength and ultimate strength were in the range of 170–320 MPa, 320–410 MPa, respectively, and the corresponding true strain (at fracture) was in the range of 10–22%.

**Table 2:** The values of the compressive mechanical properties of the sintered Mg–15Gd–1Zn bulks.

Temperature (^o^C)	Pressure (MPa)	Time (min)	Yield stress (MPa)	Ultimate stress (MPa)	True strain to failure
430	40	5	306	405	0.1
		10	201	341	0.18
	45	5	266	421	0.17
		10	189	282	0.1
	50	5	316	388	0.14
450	40	5	298	374	0.13
	45	5	310	411	0.16
		10	299	382	0.13
	50	5	291	366	0.13
470	45	3	243	411	0.21
		5	260	410	0.17
	50	5	258	378	0.15

**Figure 10:**
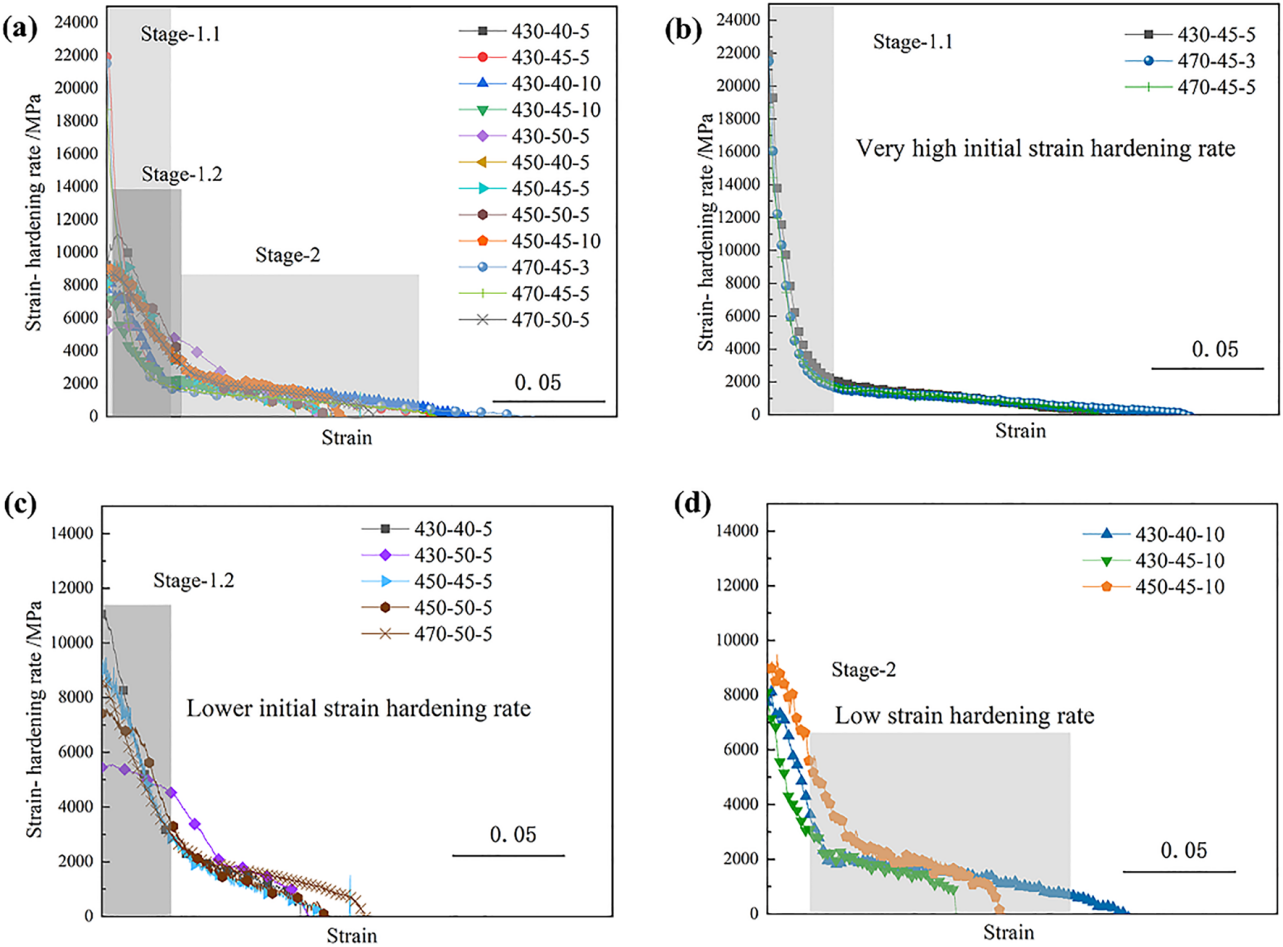
Strain hardening rate vs. true strain at different sintering processes: (**a)** all strain hardening rate curves; (**b)** the first kind; (**c)** the second kind; (**d)** the third kind hardening behavior.

The strain-hardening rate was analyzed under various sintering conditions, as shown in Fig. 10. All the strain hardening rate curves show the typical two-section hardening behaviors. The first section was the rapid descent after the initial plastic deformation, and the hardening rate rapidly decreased from tens of thousands to only about 2000–3000 MPa (including Stage-1.1 and Stage-1.2). Stage-1.1 shows a very high initial strain hardening rate at the beginning of plastic deformation, and stage-1.2 was related to a lower initial strain hardening rate and slower decrease with increase of the strain than that of stage-1.1. The second section was about the stable and weak strengthening stage, stage-2, at the latter deformation process with rising of true stress, and the hardening rate is just less than 1000 MPa.

The strain-hardening rate curves could be divided into three kinds, according to the different features of the strain-hardening rate. The first one was a high initial strain-hardening rate at the beginning of plastic deformation but fast reduction with increase of strain, which reached up to about 20,000 MPa, like the sintering of 430 °C −45 MPa-5 min, 470 °C −45 MPa-3 min, and 470 °C −45 MPa-5 min, as shown in Fig. 10b. The second one was about the lower initial strain-hardening rate and slight reduction with increasing of stain. Their initial hardening rates were about 5000–10,000 MPa. Meantime, they gradually decreased to about 3000 MPa or more, especially for the sintered bulk of 450 °C −50 MPa-5 min and 47 0 °C −50 MPa-5 min, as shown in Fig. 10c. The third kind was about the smallest hardening effect during the plastic deformation, and the values were only about 2000 MPa at the beginning of the latter deformation, such as the sintering of 430 °C −50 MPa-5 min, as shown in Fig. 10d.

### The strengthening mechanism analysis of the sintered alloys

**Figure 11:**
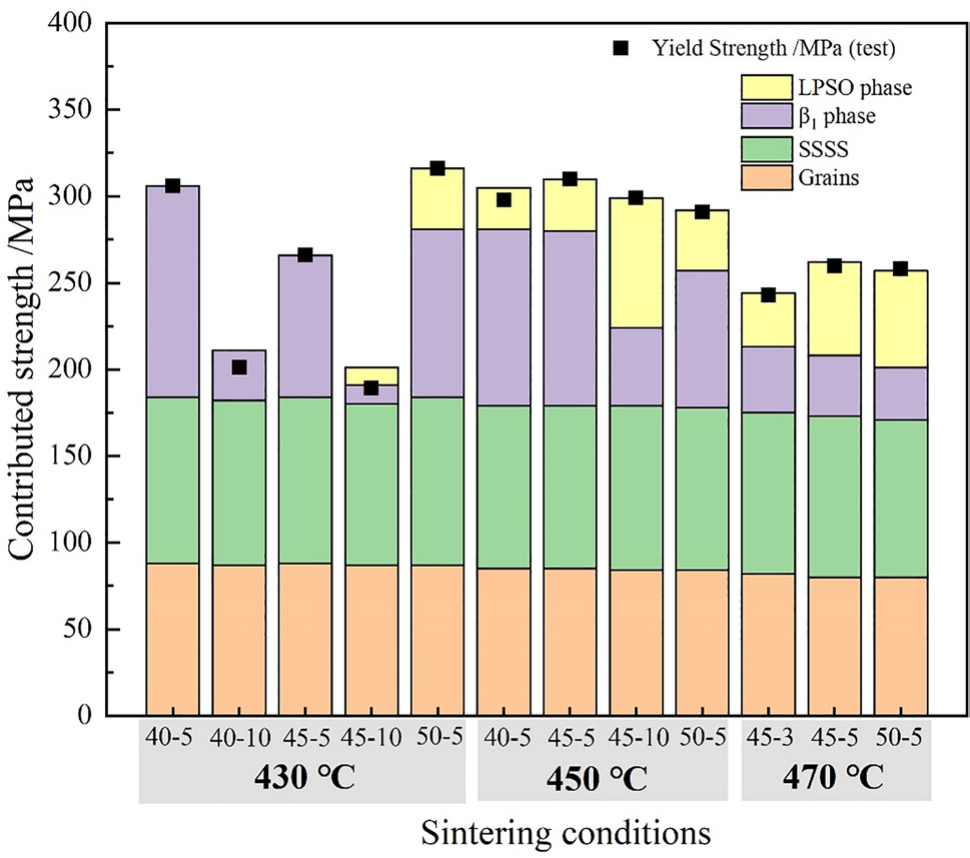
The yield stress contribution of the sintered samples, including four parts: refining grains, SSSS, β_1_ phase and LPSO phase. The calculation process was based on Hall–Petch relation, solid-solution strengthening relation, and Orowan relation^25,35^, the detailed information was shown in Table S3. The black square dots was the actual tested yield stress.

Figure 11 shows the strengthening contribution to yield stress analysis basing the quantitative contribution analyzing method for the sintered alloys, which contained three parts: refining grains strengthening, solid solution strengthening and second-phases strengthening. For each sample, both refining grains strengthening and solid solution strengthening were basically similar, while the second phases, including β_1_ phase and LPSO phase, have distinctly different contribution to the yield stress. β_1_ phase (less than 100 nm) has significantly strengthening the matrix, such as sintering 430/450 °C −40/45 MPa-5 min. When they were sintered long time at higher temperature, the LPSO phase strengthening contributed a lot, along with a decreasing β_1_ phase strengthening, due to the size coarsening of β_1_ phase (larger than 100 nm, and even 200 nm).

## Conclusion

The lower temperature sintering rapid solidification (RS) Mg–15Gd–1Zn (wt.%) ribbons were systematically studied in this work. The synergistic strengthening microstructure was investigated containing two second-phases and supersaturated solid solution (SSSS), which was hard to achieve via the conventional plastic deformation processes. The following conclusions can be drawn:

1. Rapid solidification ribbons preparation and lower temperature sintering were the keys to obtain effective solid solution strengthening.
2. Grains refinement and multiple second-phases were acquired during the sintering process, β_1_ phase with less than 100 nm and lamellar LPSO phase, could realize synergetic strengthening in the sintered Mg–Gd–Zn alloy.
3. The growth of the β_1_ phase is related to the changing of the peak intensity of (111) and (220), while they have the same changing with the prismatic planes of the Mg matrix, like (100)α. The sintering temperature has a particular effect: the higher temperature reduces the relative intensity of (111)_β1_ and (220)_β1_. The sintering time and pressure have little effect on the orientation changing of the β1 phase.
4. The strengthening mechanism analysis show a higher initial strengthening effect could be obtained when they contained a high content of β_1_ phase particles with less than 100 nm. In contrast, the slightly higher strengthening effect would achieve during the plastic deformation stage when it contained a certain content of β_1_ phase particles larger than 200 nm.

## Data availability 

The raw data of this study was displaced in Supplementary materials.

## Supplementary Information

Supplementary Information.
