# Supplementary material for: Microstructure and mechanical properties of the Mg–Gd–Zn alloy prepared by sintering of rapidly-solidified ribbons
Source: Sci Rep. 2022 Jun 29;12:11003. doi: 10.1038/s41598-022-14753-2 (PMC9243015; doi:10.1038/s41598-022-14753-2)
Supplement: Supplementary file 1 — Supplementary Information. [file 41598_2022_14753_MOESM1_ESM.doc]

**Table S1.** the origin relative density of sintering bulks

| **Temperature/^o^C** | **Pressure/MPa** | **Time/min** | **Relative density** |
| --- | --- | --- | --- |
| 430 | 40 | 3 | 0.927 (<0.98) |
|  |  |  | 0.926 |
|  |  |  | 0.925 |
|  |  | 5 | 0.998 |
|  |  |  | 0.997 |
|  |  |  | 0.996 |
|  |  | 10 | 0.997 |
|  |  |  | 0.998 |
|  |  |  | 0.997 |
|  | 45 | 3 | 0.988 |
|  |  |  | 0.99 |
|  |  |  | 0.986 |
|  |  | 5 | 0.994 |
|  |  |  | 0.995 |
|  |  |  | 0.994 |
|  |  | 10 | 0.999 |
|  |  |  | 0.998 |
|  |  |  | 0.999 |
|  | 50 | 3 | - - |
|  |  |  | - - |
|  |  |  | - - |
|  |  | 5 | 0.997 |
|  |  |  | 0.999 |
|  |  |  | 0.995 |
|  |  | 10 | 1.003 (Melted) |
|  |  |  | 1 |
|  |  |  | 1.005 |
| 450 | 40 | 3 | 0.945(<0.98) |
|  |  |  | 0.947 |
|  |  |  | 0.943 |
|  |  | 5 | 0.999 |
|  |  |  | 0.998 |
|  |  |  | 0.999 |
|  |  | 10 | 1.008 (Melted) |
|  |  |  | 1.002 |
|  |  |  | 1.005 |
|  | 45 | 3 | 0.994 |
|  |  |  | 0.997 |
|  |  |  | 0.991 |
|  |  | 5 | 0.996 |
|  |  |  | 0.997 |
|  |  |  | 0.993 |
|  |  | 10 | 0.998 |
|  |  |  | 0.999 |
|  |  |  | 0.999 |
|  | 50 | 3 | - - |
|  |  |  | - - |
|  |  |  | - - |
|  |  | 5 | 0.998 |
|  |  |  | 0.996 |
|  |  |  | 0.999 |
|  |  | 10 | 1.010 (Melted) |
|  |  |  | 1.03 |
|  |  |  | 1.01 |
| 470 | 40 | 3 | 0.954(<0.98) |
|  |  |  | 0.951 |
|  |  |  | 0.957 |
|  |  | 5 | - - |
|  |  |  | - - |
|  |  |  | - - |
|  |  | 10 | 1.009 (Melted) |
|  |  |  | 1.01 |
|  |  |  | 1 |
|  | 45 | 3 | 0.993 |
|  |  |  | 0.991 |
|  |  |  | 0.995 |
|  |  | 5 | 0.995 |
|  |  |  | 0.992 |
|  |  |  | 0.998 |
|  |  | 10 | 1.009 (Melted) |
|  |  |  | 1 |
|  |  |  | 1.011 |
|  | 50 | 3 | - - |
|  |  |  | - - |
|  |  |  | - - |
|  |  | 5 | 0.998 |
|  |  |  | 0.998 |
|  |  |  | 0.999 |
|  |  | 10 | 1.010(Melted) |
|  |  |  | 1 |
|  |  |  | 1.012 |

**Table S2**. The origin data of second-phase particles distribution and frequency in all sintering alloys

|  |  | 430-40-5 | 430-40-5 | 430-40-10 | 430-40-10 | 430-45-5 | 430-45-5 | 430-45-10 | 430-45-10 | 430-50-5 | 430-50-5 |
| --- | --- | --- | --- | --- | --- | --- | --- | --- | --- | --- | --- |
| **Size distribution/nm** | **Mean size /nm** | **Mean size /nm** | **Frequency** | **Mean size /nm** | **Frequency** | **Mean size /nm** | **Frequency** | **Mean size /nm** | **Frequency** | **Mean size /nm** | **Frequency** |
| ＜50 | ＜50 | 41.16654 | 0.07831 | 67.005 | 0.01918 | 45.17 | 0.0784 |  |  | 44.20889 | 0.11111 |
| 50-79.9 | 50-80 | 66.04167 | 0.37952 | 94.57525 | 0.09592 | 70.34 | 0.2943 | 74.62 | 0.00671 | 65.1876 | 0.26749 |
| 80-109.9 | 80-110 | 94.35513 | 0.34639 | 127.11333 | 0.14388 | 90.67 | 0.1458 | 98.76417 | 0.04027 | 94.10231 | 0.21399 |
| 110-139.9 | 110-140 | 122.34125 | 0.12048 | 154.53742 | 0.14868 | 122.36 | 0.2171 | 125.2012 | 0.16779 | 127.15714 | 0.08642 |
| 140-169.9 | 140-170 | 153.06294 | 0.0512 | 184.52137 | 0.1223 | 155.05 | 0.0532 | 155.53306 | 0.16443 | 150.95188 | 0.06584 |
| 170-199.9 | 170-200 | 187.856 | 0.01506 | 223.8076 | 0.17986 | 186.305 | 0.0542 | 186.305 | 0.13423 | 188.64111 | 0.03704 |
| 200-249.9 | 200-250 | 220.54667 | 0.00904 | 276.56192 | 0.1247 | 216.64 | 0.0521 | 223.62322 | 0.19799 | 227.25917 | 0.04938 |
| 250-299.9 | 250-300 |  | -- | 324.15632 | 0.09113 | 255.66 | 0.0367 | 275.67514 | 0.11745 | 277.01667 | 0.06173 |
| 300-349.9 | 300-350 |  | -- | 375.66083 | 0.02878 | 322.84417 | 0.0205 | 322.84417 | 0.08054 | 326.32583 | 0.04938 |
| 350-399.9 | 350-400 | -- | -- | 423.82667 | 0.01439 | 370.06 | 0.0343 | 368.0625 | 0.05369 | 380.43714 | 0.02881 |
| 400-449.9 | 400-450 | -- | -- | 479.44857 | 0.01679 | 444.16 | 0.0134 | 434.16 | 0.01342 | 411.64667 | 0.01235 |
| 450-499.9 | 450-500 | -- | -- | 520.49667 | 0.00719 |  |  | 477.955 | 0.01342 | 479.38333 | 0.01235 |
| 500-599.9 | 500-600 | -- | -- | 621.72 | 0.0048 |  |  | 527.795 | 0.00671 | 598.92 | 0.00412 |
| 600-699.9 | 600-700 | -- | -- | -- | -- |  |  | 658.5 | 0.00336 | -- | -- |
| 700-799.9 | 700-800 | -- | -- | -- | -- | -- | -- | -- | -- | -- | -- |
| 800-899.9 | 800-900 | -- | -- | -- | -- | -- | -- | -- | -- | -- | -- |
| 900-999.9 | 900-1000 | -- | -- | 1161.38 | 0.0024 | -- | -- | -- | -- | -- | -- |
| ＞1000 | ＞1000 | -- | -- | -- | -- | -- | -- | -- | -- | -- | -- |
|  |  | -- | -- |  |  | -- | -- | -- | -- | -- | -- |
|  |  | 450-40-5 | 450-40-5 | 450-45-3 | 450-45-3 | 450-45-5 | 450-45-5 | 450-45-10 | 450-45-10 | 450-50-5 | 450-50-5 |
| **Size distribution/nm** | **Mean size /nm** | **Mean size /nm** | **Frequency** | **Mean size /nm** | **Frequency** | **Mean size /nm** | **Frequency** | **Mean size /nm** | **Frequency** | **Mean size /nm** | **Frequency** |
| ＜50 | ＜50 | 40.14186 | 0.32177 | 42.52632 | 0.09005 | 37.8125 | 0.19608 | 38.49 | 0.00279 | 41.19229 | 0.16552 |
| 50-79.9 | 50-80 | 59.66778 | 0.25552 | 65.68518 | 0.40284 | 64.00182 | 0.10784 | 69.31818 | 0.03064 | 63.7328 | 0.43103 |
| 80-109.9 | 80-110 | 89.98125 | 0.05047 | 92.10979 | 0.22749 | 96.96421 | 0.09314 | 94.77552 | 0.08078 | 92.32078 | 0.17586 |
| 110-139.9 | 110-140 | 118.66846 | 0.04101 | 122.428 | 0.11848 | 127.5395 | 0.09804 | 126.14571 | 0.13649 | 125.80333 | 0.05172 |
| 140-169.9 | 140-170 | 151.39125 | 0.02524 | 156.40842 | 0.09005 | 156.81696 | 0.11275 | 156.16403 | 0.1727 | 152.725 | 0.04138 |
| 170-199.9 | 170-200 | 182.27667 | 0.00946 | 182.895 | 0.04739 | 185.725 | 0.09804 | 182.46163 | 0.18106 | 184.64667 | 0.02069 |
| 200-249.9 | 200-250 | 218.54636 | 0.0347 | 233.78 | 0.01422 | 218.85 | 0.12255 | 223.13943 | 0.19499 | 224.66667 | 0.02069 |
| 250-299.9 | 250-300 | 285.72083 | 0.03785 |  |  | 269.17313 | 0.07843 | 272.20676 | 0.10306 | 272.69167 | 0.02069 |
| 300-349.9 | 300-350 | 321.76182 | 0.0347 |  |  | 320.56667 | 0.05882 | 322.06471 | 0.04735 | 327.32833 | 0.02069 |
| 350-399.9 | 350-400 | 375.67125 | 0.02524 | 381.08 | 0.00948 | 372.875 | 0.01961 | 376.30125 | 0.02228 | 389.015 | 0.0069 |
| 400-449.9 | 400-450 | 424.10182 | 0.0347 |  |  |  | 0 | 414.40857 | 0.0195 | 428.22 | 0.01034 |
| 450-499.9 | 450-500 | 471.22 | 0.05047 |  |  | 472.44 | 0.0049 | 494.59 | 0.00836 | 461.52 | 0.0069 |
| 500-599.9 | 500-600 | 538.41313 | 0.05047 |  |  |  | 0 |  |  | 547.316 | 0.01724 |
| 600-699.9 | 600-700 | 671.05222 | 0.02839 |  |  | 631.25 | 0.0098 |  |  | 674.91 | 0.0069 |
| 700-799.9 | 700-800 | -- | -- |  |  |  | 0 |  |  | -- | -- |
| 800-899.9 | 800-900 | -- | -- |  |  |  | 0 |  |  | 881.3 | 0.00345 |
| 900-999.9 | 900-1000 | -- | -- |  |  |  | 0 |  |  | -- | -- |
| ＞1000 | ＞1000 | -- | -- |  |  |  |  |  |  |  |  |
|  |  | -- | -- |  |  |  |  |  |  |  |  |
|  |  | **470-45-3** | **470-45-3** | **470-45-5** | **470-45-5** | **470-50-5** | **470-50-5** |  |  |  |  |
| **Size distribution/nm** | **Mean size /nm** | **Mean size /nm** | **Frequency** | **Mean size /nm** | **Frequency** | **Mean size /nm** | **Frequency** |  |  |  |  |
| ＜50 | ＜50 | 30 | 0.00325 | 34 | 0.00432 |  |  |  |  |  |  |
| 50-79.9 | 50-80 | 60.76923 | 0.04221 | 62.4592 | 0.07241 | 72.91 | 0.00627 |  |  |  |  |
| 80-109.9 | 80-110 | 92.30769 | 0.12662 | 95.3769 | 0.12662 | 99.97385 | 0.04075 |  |  |  |  |
| 110-139.9 | 110-140 | 122.63158 | 0.12338 | 121.6258 | 0.12338 | 126.75955 | 0.06897 |  |  |  |  |
| 140-169.9 | 140-170 | 150.93023 | 0.13961 | 154.9453 | 0.0778 | 154.06316 | 0.11912 |  |  |  |  |
| 170-199.9 | 170-200 | 180 | 0.13312 | 183.21 | 0.11312 | 184.25417 | 0.11285 |  |  |  |  |
| 200-249.9 | 200-250 | 223.75 | 0.12987 | 223.07 | 0.17987 | 225.69718 | 0.22257 |  |  |  |  |
| 250-299.9 | 250-300 | 265.67568 | 0.12013 | 270.0435 | 0.1112 | 272.19941 | 0.10658 |  |  |  |  |
| 300-349.9 | 300-350 | 317.5 | 0.07792 | 327.512 | 0.09792 | 324.38138 | 0.09091 |  |  |  |  |
| 350-399.9 | 350-400 | 365.83333 | 0.03896 | 365.2324 | 0.05326 | 369.45313 | 0.05016 |  |  |  |  |
| 400-449.9 | 400-450 | 414.28571 | 0.02273 | 434.2871 | 0.02065 | 427.84333 | 0.02821 |  |  |  |  |
| 450-499.9 | 450-500 | 470 | 0.02273 | 475.3245 | 0.01021 | 475.85857 | 0.02194 |  |  |  |  |
| 500-599.9 | 500-600 | 537.5 | 0.01299 | 539.4564 | 0.00599 | 541.53105 | 0.05956 |  |  |  |  |
| 600-699.9 | 600-700 | 690 | 0.00325 | 696.4538 | 0.00325 | 654.65 | 0.01881 |  |  |  |  |
| 700-799.9 | 700-800 | -- | -- |  |  | 762.64714 | 0.02194 |  |  |  |  |
| 800-899.9 | 800-900 | -- | -- |  |  | 831.6375 | 0.01254 |  |  |  |  |
| 900-999.9 | 900-1000 | -- | -- |  |  | 928.65 | 0.01567 |  |  |  |  |
| ＞1000 | ＞1000 | 1330 | 0.00325 |  |  | 1499.87 | 0.00313 |  |  |  |  |

Table S3 shows the yield stress contribution parts, firstly, the mean grains-sizes were about 4.6, 5.1, and 5.8 μm. The values of the fine-grain strengthening were about 87, 84, and 80 MPa, respectively, according to the Hall-Petch relatio, and their differences were relatively small. Secondly, the content of Gd was about 2 at.% (12 wt.%), which implies that a nearly same solid solution strengthening effect in all sintering bulks, the strengthening contribution was about 91-97 MPa. For second phases, the size and content are the major factors that affect the strength, the smaller particles, and the higher strengthening effect, especially for the particles less than 100 nm. Like the β_1_ phase, the stress contribution was about (11-122) MPa, and (0-75) MPa for the LPSO phase, under the various sintering conditions.

**Table S3.** Stress contributions of different strengthening methods for the sintering bulks

| **Sintering alloys** | **Refining grains /MPa** | **SSSS /MPa** | **Second phase /MPa** | |
| --- | --- | --- | --- | --- |
|  |  |  | **β_1_ phase** | **LPSO phase** |
| Basis of calculation | *k*·d^-1/2^ | The upper limit was: 54·Gd at.%+ 2·Zn at.% | 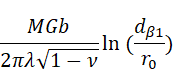 | 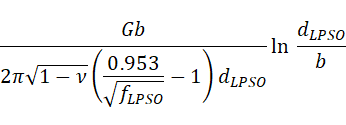 |
|  | 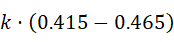 |  | (899-1091)·(0.03-0.15) | 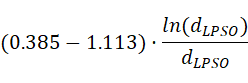 |
| The estimated values* | 80-88 | 91-97 | 11-122 | 0-75 |

where the *k* represents the Hall-Petch slope, *d* represents the mean grains size, M denotes the Taylor factor. G is the shear modulus of the alloy matrix, *b* denotes the magnitude of the Burgers vector of the slip dislocations,
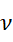
denotes the Poisson’s ration, and r_0_ denotes the core radius of the dislocations. *d_β1_* denotes the mean precipitate diameter, λ denotes the corresponding effective inter-precipitate spacing; *f*_LPSO_ means the volume ratio of LPSO in the sintering alloy, d_LPSO_ indicates the mean planar diameter of LPSO phase.


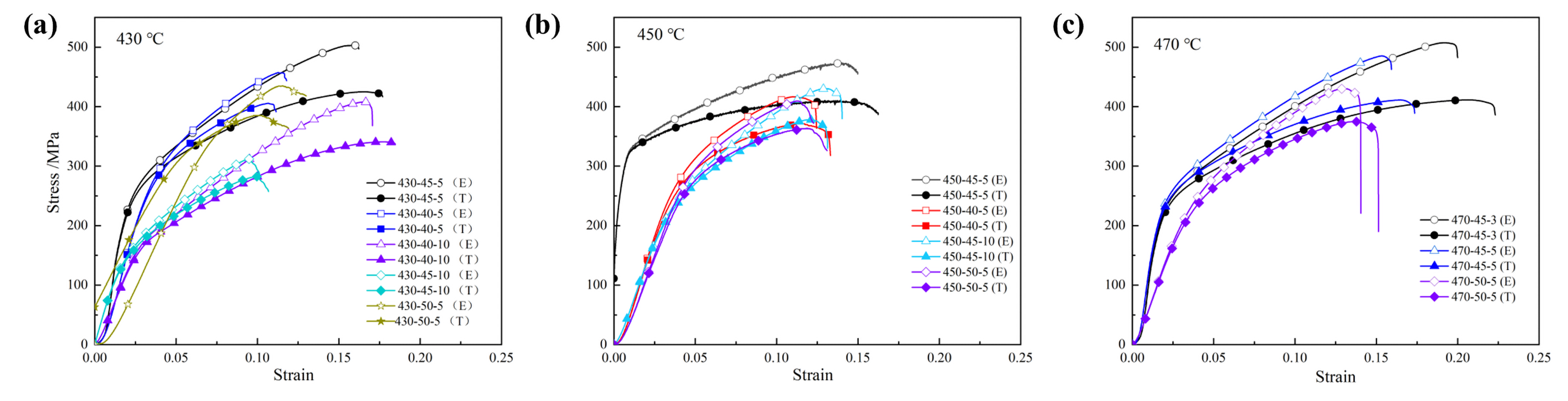


**Figure S1.** The original engineering stress vs strain curves and true stress vs strain curves containing elastic and plastic stages: **a** for sintering 430 ^o^C; **b** for sintering 450 ^o^C; **c** for sintering 470 ^o^C; E means engineering, and T means true stress curve.
